# Faith-based health screenings for Marshallese adults living in the Republic of the Marshall Islands: Study design and results

**DOI:** 10.3389/fpubh.2023.1075763

**Published:** 2023-03-28

**Authors:** Jennifer A. Andersen, Brett Rowland, Gail O'Connor, Williamina Ioanna Bing, Sheldon Riklon, Philmar Mendoza-Kabua, Pearl A. McElfish

**Affiliations:** ^1^College of Medicine, University of Arkansas for Medical Sciences Northwest, Springdale, AR, United States; ^2^Office of Community Health and Research, University of Arkansas for Medical Sciences Northwest, Springdale, AR, United States; ^3^Iakwe Home Health LLC, Honolulu, HI, United States

**Keywords:** Republic of the Marshall Islands, Native Hawaiian and Pacific Islanders, type 2 diabetes, hypertension, community-based participatory research, faith-based organizations

## Abstract

**Introduction:**

Striking health disparities exist in the Republic of the Marshall Islands (RMI). The RMI has one of the highest age-adjusted type 2 diabetes mellitus (T2DM) rates in the world (23.0%) compared to global (9.3%) and United States (US; 13.3%) rates. We conducted health screenings including clinical indicators of T2DM and hypertension among Marshallese in the RMI.

**Methods:**

Screenings were conducted at 20 churches on Majuro Atoll. Participants completed questionnaires and biometric data collection assessing glycated hemoglobin (HbA1c), blood pressure, and body mass index.

**Results:**

Screenings included 528 participants and showed a high prevalence of T2DM, obesity, and hypertension. One-third of participants were referred to the non-communicable disease clinic. The percent of adults in this study with T2DM-indicative HbA1c (48.5%) is higher than observed at the national level (23.0%).

**Discussion:**

Results highlight the need for non-communicable disease-related programs in the RMI.

## Introduction

The Republic of the Marshall Islands (RMI) is part of the United States (US) Affiliated Pacific Islands (USAPI) through a Compact of Free Association (COFA) (1). The RMI is made up of 29 atolls and five isolated islands in the North Pacific Ocean, about halfway between Hawaii and Australia, with a population of ~80,000 (2). Although research in the RMI is limited, the available data demonstrates striking health disparities including a high rate of type 2 diabetes mellitus (T2DM) (3–6). The International Diabetes Foundation has ranked the RMI with one of the highest age-adjusted T2DM rates in the world (23.0%) (7) compared to lower rates in the US (13.3%) and globally (9.3%) (7, 8).

Health disparities in the RMI are linked to historical trauma experienced by Marshallese, resulting from testing of nuclear weapons on the atolls by the US (9, 10). Between 1946 and 1958, the US military tested numerous nuclear weapons, resulting in the exposure of Marshallese to significant levels of radiation (9, 10). Due to the contamination of the RMI from nuclear testing, the Marshallese transitioned from a diet sourced through local farming and fishing to a diet reliant on highly processed commodity foods imported from the continental US and a more sedentary lifestyle (9–13). Further, American scientists conducted research on the Marshallese people in an attempt to understand the effects of nuclear fallout; however, this research was conducted without consideration of language differences or informed consent, creating distrust in outside researchers (9, 10).

The long-term objective of the study team is to understand and address the epidemic of T2DM in the RMI in collaboration with the Ministry of Health and non-governmental organizations in the RMI. The purpose of this study was to conduct health screenings as part of a needs assessment that included clinical health indicators of T2DM and hypertension among Marshallese living in the RMI, as well as diabetes self-management knowledge and behaviors for Marshallese previously diagnosed with T2DM. The main aims of the health screenings were to: (1) conduct a needs assessment to understand the current prevalence and severity of T2DM in faith-based organizations in the RMI; and (2) identify the barriers to T2DM treatment and self-management in the RMI. The findings from the health screenings will be utilized to inform future T2DM and other non-communicable disease related programs in the RMI. Health counseling was provided to all participants, and referrals to health care providers were arranged for those participants with screening results out of the normal/healthy range.

## Study design

### Community-based participatory research

This study utilized a community-based participatory research (CBPR) approach, which promotes equitable and ethical research (14–17). The use of a CBPR approach is important given the historical trauma experienced by the Marshallese people, including the nuclear weapons testing conducted in the RMI by the US military and the resulting unethical research on Marshallese exposed to nuclear fallout (9). CBPR engages community partners, honors their unique contributions at all stages of research, and ensures Marshallese cultural knowledge informs the process (18).

### Community partners

A community advisory board that included seven members from our community partners in the RMI led the assessment. Community partners included the RMI Ministry of Health and Human Services (MOHHS), the Marshallese Educational Initiative (MEI), and Kora In Jiban Lolorjake Ejmour (KIJLE; approximately translated to “Women for Health”). The MOHHS has been designated by the RMI's Constitution as the state health agency and is vital to researchers appropriately implementing research activities in the RMI (19). The study team has built a strong working relationship with MOHHS and has full time staff with a dedicated office space in MOHHS. MOHHS worked closely with the study team to develop the study protocol and received weekly updates on study activities and progress. The study team collaborated with the MOHHS non-communicable disease (NCD) team to provide counseling at each screening event for all participants. Participants who needed further care were referred to the NCD clinic with scheduled appointments provided by NCD providers at screening events.

MEI is a non-profit organization that promotes cultural, intellectual, and historical awareness of the Marshallese people; MEI is headquartered in Springdale, Arkansas with outreach in the RMI. KIJLE is a non-profit women's group, which collaborates with the MOHHS to engage the community in public health initiatives. KIJLE is important to maintaining cultural congruence during implementation as they represent the matriarchal leadership of the RMI.

### Study setting

The study was conducted in 20 churches located on Majuro Atoll. Churches play an important role in Marshallese culture; prior needs assessments in Arkansas have shown that 96.5% of Marshallese adults report regular church attendance (20). Most of the health screenings were done inside the church buildings; however, due to limited room at some locations, a few set up the health screenings at an outside location. In all cases, the study team arranged the counseling tables away from the general screening area for privacy. The study team worked with the churches to ensure rooms were available for glycated hemoglobin (HbA1c) testing to ensure the testing kits could remain temperature controlled.

### Study team

The study team was comprised of the principal investigator and several co-investigators who have prior experience conducting research studies with Marshallese participants in the US and the RMI. The study manager has 15 years of community health and research experience and is a native of the RMI. The study manager previously relocated to the RMI and was responsible for the management of all local community health workers (CHWs) who served as research staff during this study. Local CHWs and research staff completed Collaborative Institutional Training Initiative Human Subjects research training, training on Health Insurance Portability and Accountability Act of 1996 privacy requirements, blood borne pathogen safety training, trainings in biometric data collection, and study-specific trainings. At least six data collectors and one MOHHS NCD provider were present at every data collection event.

### Church and participant recruitment

CHWs and community advisory board members assisted with recruiting churches to participate in the health screenings. Additional recruitment efforts included announcements on the local radio station, through text messaging, and through invitations extended to non-participating churches who inquired about participation after hearing of the health screenings taking place elsewhere. When a church was identified as being interested in participating, the study team would meet with the pastors face-to-face to present information about the needs assessment and its recruitment goals. Once a church agreed to participate, town hall style question and answer sessions were used to inform and recruit individual participants. Additionally, the health screenings were advertised *via* postings on local websites, social media, and study flyers. Potential participants were advised of the time commitment involved in the health screening and were invited to participate in other scheduled screenings if they were unable to stay for the full screening event.

### Eligibility determination

The study team captured biometric and survey data on individuals who met the inclusion criteria and consented to participate in the study. Participant inclusion criteria included: (1) self-identified as Marshallese and (2) over 18 years of age.

### Consent

Previous research with the Marshallese community has highlighted Marshallese participants' desire for a simplified consent process and the preference for verbal discussion of study requirements (21). Therefore, as the health screenings were considered a minimal risk study, a waiver of consent documentation was requested and granted by the University of Arkansas for Medical Sciences Institutional Review Board (IRB #262557). The study information sheet was provided to participants in Marshallese, and plain language was used to increase understanding. Participants were allowed time to read or have a CHW read the information sheet to them. Participants were encouraged to ask any questions they may have had. All participants received a paper version of the study information sheet in Marshallese for their records. Participants verbally acknowledged consent prior to data collection.

### Remuneration

Remuneration was provided to all participants who consented to take part in the study. Participants were given $10 as compensation for their participation. Individuals who wanted to receive a health screening but declined the opportunity to participate in the study were provided a screening free of charge but did not receive compensation.

## Methods and analysis

### Data collection

All data collection staff had previous experience collecting biometric and survey data in the RMI. REDCap was utilized to store and manage data (22). To prevent/minimize missing data, REDCap includes a missing data report in the Quality Assurance tool which allowed for convenient quality assurance validation and monitoring, as well as prompt collection of missing data. All of the instruments used in the study were chosen collaboratively with Marshallese stakeholders and have been translated into Marshallese and validated with Marshallese participants. The research team has used these same questions with more than 1,000 Marshallese participants in multiple studies (23, 24).

### Biometric data

The biometric data collection was completed by qualified, trained research staff and was carried out using standard infection, prevention, and control practices. Waste was disposed of in a safe manner in accordance with MOHHS instructions. Biometric measures collected included HbA1c, weight, height, and blood pressure. HbA1c was collected *via* finger prick using aseptic techniques and analyzed using PTS Diagnostic's A1CNOW+ point-of-care HbA1c tests. Participants' weight (without shoes) was measured to the nearest 0.5 pound using a calibrated digital scale. Height (without shoes) was measured to the nearest 0.25 inch using a stadiometer. Body mass index (BMI) was calculated using collected height and weight [(weight in pounds^*^703)/(height in inches^2^)]. Systolic and diastolic blood pressure was measured using a digital blood pressure device with the participant seated, arm elevated, and cuff placed at approximately heart height. Categorical blood pressure (normal, pre-hypertension, stage 1 hypertension, and stage 2 hypertension) were informed by the American Heart Association standards (25). Pulse pressure was calculated by subtracting the diastolic from the systolic blood pressure value (26). All individuals who participated in the health screening received a paper copy of their biometric results.

### Survey data

After consent was provided, participants took a short 10-min survey before or after the health screening. The survey instrument included questions adapted from the Behavioral Risk Factor Surveillance System (BRFSS) survey (27). All survey documents were translated into Marshallese and were self-administered or interviewer-administered, depending on the preference and/or literacy level of the participant. Due to past findings from research with Marshallese communities in the RMI and Arkansas, the length of the survey was reduced from prior studies to ensure accurate responses and to reduce participant burden.

### Analysis

Descriptive statistics, including means and standard deviations for continuous variables and frequency and percentages for categorical variables, are used to report the results of the health screenings. Descriptive statistics are provided for demographic and biometric data. The analysis was completed using STATA 17 (28).

## Results

### Sample

Five hundred and twenty eight (528) Marshallese adults participated in the health screenings. Due to issues and delays with the supply chain (in some cases, supplies took more than a month to arrive from the US with unreliable tracking data), only 450 participants were able to have their HbA1c tested during the screenings. Table 1 presents the demographics of the sample and the results of the health screenings.

**Table 1:** Participant demographics and biometrics.

	**Obs**	***N* (Mean ±SD)**	**% of Sample**	**Range**
**Sex**	527			
Female		326	61.9	–
Male		201	38.1	–
**Education**	527			
Never attended		4	0.8	–
Elementary		98	18.6	–
Some high school		173	32.8	–
High school graduate or GED		133	25.2	–
Some college or technical school		86	16.3	–
College graduate		33	6.3	–
Age (continuous)	528	43.4 ± 15.1		18–83
**Age (categorical)**	528			
18–24		72	13.6	–
25–44		217	41.1	–
45–64		186	35.2	–
>65		53	10.0	–
HbA1c (continuous)	450	7.2 ± 2.5%		4.1–14.1%
**HbA1c (categorical)**	450			
Normoglycemic (≤ 5.6)		166	36.9	–
Pre-diabetes (5.7–6.4)		80	17.8	–
Diabetes (≥6.5)		204	45.3	–
Weight (in pounds)	525	168.3 ± 38.3		85.8–311.2
BMI	525	31.4 ± 6.8		17.2–58.8
**BMI (categorical)**	525			
Underweight		6	1.1	–
Normal weight		86	16.4	–
Overweight		135	25.7	–
Obese		298	56.8	–
Systolic blood pressure (mmHg)	528	118 ± 19		82–211
Diastolic blood pressure (mmHg)	528	73 ± 11		44–119
**Blood pressure (categorical)**	528			
Normal (<120/80)		274	51.9	–
Pre-hypertension (120–129/ <80)		191	36.2	–
Stage 1 (130–139/80–89)		43	8.1	–
Stage 2 (≥140/≥90)		20	3.8	–
Pulse pressure (mmHg)	528	45 ± 14		12–119
**Pulse pressure (categorical)**	528			
Normal		224	42.4	–
Elevated		229	43.4	–
High		75	14.2	–
**Advised to seek medical care regarding results**	526			
No		343	65.2	–
Yes		183	34.8	–

Only valid percentages shown. Percentages may not total 100 due to rounding.

SD, standard deviation; GED, graduate equivalency degree; HbA1c, hemoglobin A1c; BMI, body mass index.

HbA1c only completed for 450 participants due to supply chain issues.

### Demographics

The sample was predominately female (61.9%) with a mean age of 43.4 years (± 15.1 years). Just over one-third (35.2%) of the participants were between the ages of 45 and 64. The majority of participants either had some high school education (32.8%) or were a high school graduate (25.2%).

### Biometrics

#### HbA1c

The mean HbA1c of the sample was 7.2% (± 2.5%). The majority of the sample had an HbA1c indicative of glucose dysregulation; 17.8% had an HbA1c indicating prediabetes (5.7–6.4%) and 45.3% had an HbA1c indicating diabetes (≥6.5%).

#### Weight and BMI

The mean weight of the sample was 168.3 lbs. (± 38.3 lbs.), and the mean BMI was 31.4 ± 6.8. The majority of participants were either overweight (25.7%) or obese (56.8%).

#### Blood pressure and pulse pressure

The sample had a mean systolic blood pressure of 118 mmHg (± 19 mmHg) and a mean diastolic blood pressure of 73 mmHg (± 11 mmHg). Just over a third (36.2%) had blood pressure indicative of prehypertension, 8.1% had blood pressure indicative of stage 1 hypertension, and 3.8% had blood pressure indicative of stage 2 hypertension.

The mean pulse pressure of the sample was 45 mmHg (± 14 mmHg). Forty-three (43.4%) percent had an elevated pulse pressure (40–49 mmHg), and 14.2% had a high pulse pressure (≥50 mmHg).

#### Referrals to medical care

Over a third (34.8%) of participants were referred to the NCD clinic with scheduled appointments with a health care provider.

### Dissemination plan

Throughout previous work with the Marshallese community, the study team has found that individual in-person meetings, as well as church meetings, town hall meetings, using infographics, and using plain language summaries, are the culturally preferred methods for dissemination of study results (29). Individual participant results were shared with participants during the diabetes health screening events.

Study staff will provide a summary of the results back to the Marshallese community utilizing existing community partnerships. Culturally and linguistically appropriate infographics and summaries will be created and used as flyers and posters to be distributed or displayed at community events. Flyers and/or posters will also be available in a digital format for posting on Facebook or other social media platforms.

To ensure participant confidentiality is maintained, aggregated de-identified results will be shared with the congregations at participating churches. Aggregated de-identified results may also be shared in town hall style events hosted by community partners; town hall meetings will be announced through social media, newspaper, and radio. A summary of the results will be provided in a formal report and presentation to the RMI MOHHS. Additionally, results of this study will be used for academic presentations, posters, or publications.

## Discussion

In this study, Marshallese adults from 20 churches on Majuro Atoll in the RMI were invited to participate in diabetes health screenings. This report describes the recruitment strategies and study protocol and provides the results of the biometric data. The data from our study will inform our future research addressing health disparities in the RMI, especially those related to cardiometabolic diseases including diabetes and hypertension.

The results of the screenings show a high prevalence of diabetes, obesity, and hypertension among the participants in the study. The percent of screened adults with an HbA1c indicative of diabetes in this study (45.3%) is even higher than the diabetes prevalence observed at the national level (23.0%) (7). This is also higher than the proportion found in similar health screenings for Marshallese adults conducted in northwest Arkansas (38.4%) (24). These results provide evidence of the health disparities faced by the Marshallese living in the RMI and highlight the need for further diabetes and other non-communicable disease-related programs in the RMI. Although the rate of non-communicable disease among the participants in the health screening study was high, we were able to assist over 180 people in setting up appointments with non-communicable disease providers.

Historical trauma experienced by Marshallese, resulting from testing of nuclear weapons on the atolls by the US, has long been linked to the health disparities the small island nation faces today (9, 10). Between 1946 and 1958, the US military tested numerous nuclear weapons, resulting in the exposure of Marshallese to significant levels of radiation (9, 10). Following these tests, American scientists conducted research on the Marshallese population in an attempt to understand the effects of nuclear fallout without any consideration of language differences or informed consent, creating distrust in outside researchers (9, 10). Given the long history between the US and the RMI, it is important for our research team to develop a trusting relationship with the Marshallese community.

In 2011, members of the research team became increasingly aware of the significant health disparities of the Marshallese community in northwest Arkansas and began meeting with Marshallese community members, Marshallese community organizations, and Marshallese churches with the goal of setting a community-driven research agenda using a CBPR approach (30). The research team conducted field work to gain a better understanding of Marshallese history and culture, and community members were invited to share their history, stories, and perspectives on health and research (30). Members of the Marshallese community were asked to join with the research team to review secondary data from the Census, Behavioral Risk Factor Surveillance System (BRFSS), Arkansas Department of Health Vital Records, and needs assessments conducted in 2004 and 2010 by the local hometown health coalition and community foundation (30). Through this work, Marshallese community members asked the research team to focus their efforts on addressing the high rates of T2DM in the community.

As part of developing the work on T2DM in Arkansas (later adapted for the RMI setting), the lead investigator brought together a diverse interprofessional research team and engaged 31 Marshallese community stakeholders, including patients, family members, and health care providers to select and adapt an appropriate intervention to address the high rates of T2DM with community support. The Marshallese stakeholders included patients with T2DM, family members of people with T2DM, and community health care providers. Representatives from Marshallese community-based organizations, the Marshallese Consulate, and Marshallese churches were included and represented patients, caregivers, and their organizations. Discussions were conducted both in English and in Marshallese and a bilingual translator provided interpretation. The use of the Marshallese language increased comfort and shifted the power of gaining knowledge and sharing information to the Marshallese stakeholders. Using the native language was important both to the research team and to the Marshallese stakeholders given the historical trauma experienced by Marshallese people and the distrust in American scientists it created. Further, as part of our efforts to build trust with the Marshallese community in Arkansas, we included a native Marshallese physician, Marshallese nurses, and additional Marshallese staff as part of the research team. At the urging of the Arkansas-based Marshallese stakeholders, we have worked diligently to bring our efforts to address T2DM to the RMI. Overall, because of the long-standing relationship with, and support from, the Marshallese community in Arkansas, Marshallese community organizations, and the RMI MOHHS, we have been able to successfully adapt our studies and health screenings to benefit Marshallese living in the RMI; without the support and involvement of Marshallese people, these efforts would not be possible. These relationships have been, and will continue to be, a vital part of our work with the Marshallese community in the US and the RMI; this study is one of several our research team has conducted in partnership with Marshallese communities in the US and the RMI dating back to the original conversations in 2011 (24, 30–37).

The health screening study was instrumental in highlighting the barriers to study administration in the RMI. Biometric data collection was not without its difficulties. Due to issues and delays with the supply chain (in some cases, supplies took more than a month to arrive with unreliable tracking data), only 450 participants were able to have their glycated HbA1c tested during the screenings. Maintaining the correct temperature for the A1CNOW+ test kits was difficult in churches without air conditioning, and special care was needed to ensure ice packs and coolers were available as needed to maintain the test equipment. Electricity was not always reliable, which limits the potential screening equipment that can be utilized during studies. Further, although churches play an important role in Marshallese life, there are limitations given the sheer number of events and activities that utilize these spaces. The study team did encounter some difficulties with utilizing churches for data collection. On occasion, the start time of the health screenings were delayed or ultimately rescheduled due to needing to have the buildings unlocked or due to another event taking place at the church.

There are limitations to consider when interpreting the findings. The convenience sample limits the ability to generalize the results, and due to a lack of community-level data, no direct comparisons of the study sample can be made to the general population of Marshallese living in the RMI or abroad. The sampling method also limits comparisons to other studies. Moreover, some participants in the screenings may have been aware of potential health conditions and, therefore, may have been more likely to agree to participate in the study. Finally, we recognize BMI is a problematic measure. Marshallese participants are often uncomfortable with having research staff measure their waist and/or hip circumference, which limits the options of determining weight-related risk factors.

Despite these limitations, we were able to provide health screenings to over 500 Marshallese adults. The aims of this study were 2-fold: (1) to conduct a needs assessment to understand the current prevalence and severity of diabetes within faith-based settings in the RMI and (2) to identify the barriers to T2DM treatment and self-management among members of faith-based organizations in the RMI. The data we have collected will help us to meet these aims and will position the research team and our community partners well to address the needs of Marshallese adults in the RMI.

## Data availability statement

The deidentified data underlying the results presented in this study may be made available upon reasonable request from the corresponding author, PM, at pamcelfish@uams.edu.

## Ethics statement

This study involving human participants was reviewed and approved by the University of Arkansas for Medical Sciences Institutional Review Board. Written informed consent for participation was not required for this study in accordance with the national legislation and the institutional requirements.

## Author contributions

JA, BR, and PM: study conception and design. JA, BR, GO'C, WB, and PM: data collection. JA, BR, PM, and PM-K: analysis and interpretation of results. JA, BR, GO'C, WB, SR, PM, and PM-K: draft manuscript preparation. All authors reviewed the results and approved the final version of the manuscript.
